# What the papers say

**DOI:** 10.1093/jhps/hny051

**Published:** 2019-01-11

**Authors:** Ajay Malviya

**Affiliations:** Northumbria Healthcare NHS Foundation Trust, Regenerative Medicine—ICM, Newcastle University, 10 East Brunton Wynd, Newcastle upon Tyne, UK

The *Journal of Hip Preservation Surgery* (*JHPS*) is not the only place where work in the field of hip preservation may be published. Although our aim is to offer the best of the best, we continue to be fascinated by work that finds its way into journals other than our own. There is much to learn from it, so *JHPS* has selected six recent and topical subjects for those who seek a summary of what is taking place in our ever-fascinating world of hip preservation. What you see here are the mildly edited abstracts of the original articles to give them what *JHPS* hopes is a more readable feel. If you are pushed for time, what follows should take you no more than 10 min to read. So here goes.

## STRATEGIES FOR DEALING WITH CHONDRAL LESIONS ASSOCIATED WITH FEMOROACETABULAR IMPINGEMENT

Two studies, both from Europe, have looked at the role of scaffolds in dealing with chondral defects, one using Chondro-Gide membrane [1] and another using Chitosan-based scaffold [2].

de Girolamo *et al*. [1] from Italy, in a retrospective comparative study investigated at 8-year follow-up, the failure rate (revision rate/conversion to arthroplasty) of patients with hip chondral lesions associated with femoroacetabular impingement treated by microfracture (MFx) and autologous matrix-induced chondrogenesis (AMIC). Patients aged between 18 and 55 years, with acetabular grades III and IV chondral lesions (Outerbridge), measuring 2–8 cm^2^ operated on at least 8 years before enrollment. Exclusion criteria were rheumatoid arthritis, dysplasia or axial deviation of the femoral head. There were no arthritic lesions, Tönnis <2 or joint space of at least 2 mm. MFx was performed with an awl, and the Chondro-Gide membrane used for the AMIC procedure was placed without glue. Among 130 patients, 109 fulfilled inclusion criteria. Fifty were treated by MFx and 59 by AMIC. The mHHS significantly improved in both groups from 46 to 78 for mHHS at 6–12 months, even for lesions >4 cm^2^. From 2 to 8 years, mHHS in the AMIC group was better than in the MFx group (*P* < 0.005). This mHHS improvement in the AMIC group was maintained through the 8-year follow-up period, whereas it deteriorated after 1 year in the MFx group (*P* < 0.005). Eleven patients (22%) in the MFx group required total hip arthroplasty (THA); none in the AMIC group did. Patient acceptable symptomatic state analysis confirmed similar short-term improvement, but a significant (*P* < 0.007) degradation after 2–8 years in MFx patients.

Tahoun *et al*. [2] from Spain, looked to evaluate the functional outcome of using chitosan-based material after 2 years of follow-up. Nonarthritic nondysplastic femoroacetabular impingement patients with an acetabular chondral lesion, 18–55 years of age, were included for arthroscopic repair between May 2013 and July 2015. Full-thickness chondral defects ≥2 cm^2^ were filled with chitosan-based implant after microfractures. This study included 23 patients at a mean follow-up of 38.4 months (range: 24–50 months). The mean defect size was 3.5 ± 1.0 cm^2^, principally involving zone 2 and to a lesser extent in zones 1 and 3. Using femoroplasty, the alpha angle was corrected from a mean 70.5–44.3 (*P* = 0.00001). Significant improvement occurred comparing the pre-operative to the first-year post-operative patient-reported outcomes: *P* = 0.00001 for the NAHS, *P* = 0.00004 for the iHOT33, *P* = 0.00005 for the HOS-ADL and *P* = 0.0002 for the HOS-SSS. No statistically significant change has been observed in the patient-reported outcomes obtained at the endpoint when compared with the first-year values, except for the iHOT33, which showed further significant improvement (*P* = 0.02). Up to 91% of the patients met or exceeded the minimal clinically important difference. One patient needed total hip arthroplasty.

While we still await higher level evidence it seems that microfracture alone may not be the most appropriate procedure for these patients and using a scaffold might improve the surgical outcome from functional and joint preservation perspective.

## DOES CLINICAL OUTCOME AFTER PERIACETABULAR OSTEOTOMY DEPEND ON FEMORAL AND ACETABULAR VERSION?

Patients with acetabular dysplasia often have abnormal femoral and acetabular version. The effect of combined femoral and acetabular version on clinical outcomes after periacetabular osteotomy (PAO) for the treatment of acetabular dysplasia remains unclear. Seo *et al*. [3] from Fukuoka University, Japan set out to (i) to evaluate the association of combined femoral and acetabular version with clinical outcome after PAO and (ii) to investigate the association of femoral version independently with clinical outcome after PAO.

They retrospectively reviewed the records for 92 consecutive patients (95 hips) who had undergone PAO for the treatment of symptomatic acetabular dysplasia. The patient cohort comprised 85 females and 7 males with a mean age of 38.9 years at the time of surgery. The mean duration of follow-up was 4.8 years (range: 2.0–7.2 years). Femoral and acetabular version and the alpha angle were measured on post-operative computed tomography scans. Clinical outcomes included range of motion and the modified Harris hip score. Analysis of variance was used to investigate the effect of femoral version on clinical outcomes. Analysis of covariance was used to adjust for potential covariates.

Combined femoral and acetabular version after PAO was slightly, but significantly, correlated with post-operative flexion (*r* = 0.222; *P* = 0.031) and internal rotation in flexion (*r* = 0.326; *P* = 0.001). Patients with mild femoral version (<15°) experienced significantly less post-operative internal rotation in flexion than those with severe femoral version (>35°); however, this difference was lost after adjustment for potential covariates. There were no differences among femoral version groups (mild, moderate and severe) in terms of improvements in the clinical outcomes of pain, function and activity.

The authors concluded that combined femoral and acetabular version after PAO was significantly correlated with post-operative range of motion. Abnormality of femoral version associated with acetabular dysplasia, however, did not demonstrate any effect on the clinical outcomes of PAO.

## IS COMPUTER-ASSISTED PLANNING AND NAVIGATION SYSTEM POSSIBLE FOR PAO?

Liu *et al*. [4] from Shenzhen, China in a study carried out in collaboration with University of Bern, Switzerland have looked at models to improve acetabular orientation during PAO. While PAO is an effective approach for surgical treatment of hip dysplasia in young adults, achieving an optimal acetabular reorientation remains the most critical and challenging step. Routinely, the correct positioning of the acetabular fragment largely depends on the surgeon’s experience and is done under fluoroscopy to provide the surgeon with continuous live X-ray guidance. They have developed a system that starts with a fully automatic detection of the acetabular rim, which allows for quantifying the acetabular 3D morphology with parameters such as acetabular orientation, femoral head extrusion index (EI), lateral center-edge (LCE) angle and total and regional femoral head coverage (FHC) ratio for computer-assisted diagnosis, planning and simulation of PAO. Intraoperative navigation is conducted to implement the pre-operative plan. Two validation studies were conducted on four sawbone models to evaluate the efficacy of the system intraoperatively and post-operatively. By comparing the pre-operatively planned situation with the intraoperatively achieved situation, average errors of 0.6 ± 0.3°, 0.3 ± 0.2° and 1.1 ± 1.1° were found, respectively, along three motion directions (flexion/extension, abduction/adduction and external rotation/internal rotation). In addition, by comparing the pre-operatively planned situation with the post-operative results, average errors of 0.9 ± 0.3° and 0.9 ± 0.7° were found for inclination and anteversion, respectively. Further work is perhaps required to validate it in real life scenario, but it is a step forward in developing an accurate navigation system for PAOs.

## ROLE OF HIP ARTHROSCOPY IN PATIENTS UNDERGOING PAO FOR HIP DYSPLASIA

Femoral cam deformity is frequently present in patients with acetabular dysplasia. A reduction in joint contact stress is a proposed mechanism for the increased joint longevity following PAO for hip dysplasia. Impingement from abnormal femoral offset negatively impacts clinical outcome, but this finding has not been evaluated from a biomechanical perspective previously and a threshold for performing femoral osteochondroplasty has not been established. Computational modeling can be used to identify how this deformity affects joint mechanics. Researchers from Iowa [5] set out to identify the relationship between cam deformity and joint contact stress after PAO. They hypothesized that cam deformity is associated with an increase in peak joint contact stress after PAO.

This was a retrospective review of patients treated for hip dysplasia with PAO without femoral osteochondroplasty. Patient-specific hip models created from pre- and post-operative computed tomography (CT) scans were evaluated using discrete element analysis to determine maximum joint contact stress after PAO. Twenty hips with a post-operative increase in maximum contact stress were compared with 20 that demonstrated decreased maximum contact stress. Hips were assessed for cam deformity on cross-sectional imaging. Radiographic measures of acetabular dysplasia before and after PAO were assessed and compared with the change in maximum contact stress after PAO.

They found a moderate relationship between the change in maximum contact stress and the α angle (*r* = 0.31; *P* = 0.04), and the average α angle in the hips with increased maximum contact stress was significantly different from that in the hips with decreased joint contact stress (51 ± 11.4° versus 42 ± 5.1°; *P* = 0.04). All six hips with an α angle of >60° demonstrated increased joint contact stress.

They concluded that cam deformity is common in patients with hip dysplasia and noted that α angles of >60° were associated with increased post-operative joint contact stress. The α angle should be assessed pre-operatively, and deformity should be addressed for optimal joint mechanics after PAO. This study provides biomechanical evidence supporting surgical management of femoral cam deformity for an α angle of >60°.

## DOES HIP ARTHROSCOPY HAVE A ROLE IN PATIENTS WITH SLIPPED CAPITAL FEMORAL PHYSIS (SCFE)?

In a study done in Sheffield, UK, Balakumar *et al*. [6] aimed to compare the outcomes of arthroscopic osteoplasty with open neck osteotomy for correction of the hip impingement and improvement of hip function in children with moderate to severe healed Slipped Capital Femoral Epiphysis (SCFE).

In a retrospective analysis of the hospital hip database retrieved 187 cases of SCFE from 2006 to 2013, they compared the outcome of 12 patients who underwent open neck osteotomy and deformity correction for moderate/severe healed SCFE with 10 who underwent arthroscopic osteoplasty of the hip.

In the arthroscopy cohort, the mean age at surgery was 15.8 years (range: 13–19 years) and mean follow-up was 46.1 months (range: 33–66 months). In the neck osteotomy group, the mean age at surgery was 14.6 years (11–20 years) and mean duration of follow-up was 49 months (36–60 months). The outcomes in arthroscopic osteoplasty group versus open neck osteotomy were as follows: anteroposterior (AP) slip angle 9.2° versus 10.8° (*P* = 0.0003), lateral slip angle 44.8° versus 13.5° (*P* = 0.00001), oblique plane deformity 47.1° versus 16.7° (*P* = 0.0003), alpha angle 61.88° versus 34.6° (*P* = 0.0003), anterior offset 0 mm versus 5 mm (*P* = 0.0003), modified Harris hip score (MHHS) 75.5 versus 90 (*P* = 0.003), non-arthroplasty hip score (NAHS) 67.12 versus 92.1 (*P* = 0.002), internal rotation 20° versus 50° (*P* = 0.0002), respectively.

Both radiological and function outcomes significantly improved in the arthroscopic group, although clearly the outcomes seem to be significantly better in the open neck osteotomy group. The authors concluded that in carefully selected cases, arthroscopy could be a less invasive procedure which has desirable outcomes.

## ENDOSCOPIC TREATMENT OF SCIATIC NERVE ENTRAPMENT IN DEEP GLUTEAL SYNDROME: CLINICAL RESULTS

Deep gluteal syndrome (DGS) is characterized by compression, at extra-pelvic level, of the sciatic nerve within any structure of the deep gluteal space, with the mainstay treatment being physiotherapy, rest and injections; very few however require surgical treatment. The objective of this retrospective study from Colombia [7] was to evaluate the clinical results in a relatively large group of patients with DGS treated with endoscopic technique between 2012 and 2016 with a minimum follow-up of 12 months. The patients were evaluated before the procedure and during the first year of follow-up with the WOMAC and VAIL scale.

Forty-four operations on 41 patients (36 women and 5 men) were included with an average age of 48.4 ± 14.5. The most common cause of nerve compression was fibrovascular bands. There were two cases of anatomic variant at the exit of the nerve; compression of the sciatic nerve was associated with the use of biopolymers in the gluteal region in an isolated case. The results showed an improvement of functionality and pain measured with the WOMAC scale with a mean of 63–26 points after the procedure (*P* < 0.05). However, at the end of the follow-up one patient continued to manifest residual pain of the posterior cutaneous femoral nerve. Four cases required revision at 6 months following the procedure due to compression of the scarred tissue surrounding the sciatic nerve.

The authors concluded that endoscopic release of the sciatic nerve offers an alternative in the management of DGS by improving functionality and reducing pain levels in appropriately selected patients.
